# Buffalo Medical and Surgical Association

**Published:** 1886-03

**Authors:** 


### Society Reports.

### Buffalo Medical and Surgical Association.

*Regular Meeting, held Feb. 2, 1886.*

The President, Dr. C. G. Stockton in the chair, F. R. Campbell, Secretary. Present—Drs. Phelps, Park, Burkhardt, Brecht, Bartlett, Coakley, Barnes, Fowler, Wm. G. Ring, and Hoyer.

The minutes of the preceding meeting were read and approved.

Dr. Wm. Phelps read a paper on "Thyroidectomy," giving a report of a successful operation performed by himself three years ago.

The patient was a married woman, 25 years of age. The gland began to increase in size two years before, shortly after an injury of the neck which she had sustained. The gland increased in size most rapidly during her pregnancies, finally affecting her breathing, and producing loss of voice. Dr. Phelps found that the isthmus or middle lobe of the gland was most enlarged, but the lateral lobes were also affected. He diagnosed the case as multilocular cystic goitre, and considered the case a favorable one for operation.

The operation was performed in connection with Drs. L. P. Dayton and Tremaine at the patient's home. An incision was made in the median line running from the upper part of thyroid cartilage to the sternum. The tumor in its sheath immediately appeared through the incision. Before opening the sheath, the inferior and superior thyroid arteries were ligated. Dr. Phelps emphasized the importance of ligating these vessels before going further with the operation. A neglect of this step was the common error committed by the older operators.

The sheath was then opened and nearly the whole gland removed. The operation was practically bloodless, not more than one ounce of blood being lost. The wound was treated antiseptically. Two days after the operation, the patient's temperature reached 104°. This was not due to surgical fever, but was produced by an attack of acute bronchitis, caused by exposure previous to the operation. The patient made a complete recovery, and is now in the best of health. There was no indication of the condition of myxædema described by Charcot, nor have the mental faculties been impaired in any way. Those sequelæ, he thought, had possibly been avoided on account of his being obliged to leave a small portion of the gland in situ.

The essayist quoted numerous authorities opposed to this operation, and showed that their greatest objections could be easily overcome, as was now done by the European surgeons, who frequently extirpate the thyroid gland, sometimes for cosmetic ends alone.

DISCUSSION.

Dr. Park congratulated Dr. Phelps on the success of his operation, saying that a surgeon who would have attempted, ten years ago, to remove the thyroid gland, would have been looked upon as bold even to rashness. At present, however, the operation was frequently performed. Billroth has already operated on 103 cases, Kocher on 250, while many other Continental surgeons have had numerous cases. There were three things which helped to render the operation safe at the present time: (1) antisepsis, (2) the hemostatic forceps, and (3) the cat-gut ligature. The principal danger to be avoided was injury of the nerves.

Dr. Park thought that the probabilities of myxædema following extirpation of the thyroid gland had been over-estimated. In Switzerland, cretinism frequently followed the operation; but this was attributed to the disease beginning before the operation was performed. In other parts of Europe, cretinism was rarely observed in connection with the enlarged thyroid. Dr. Park reported two cases, recently operated on by himself at the Buffalo General Hospital:

Case I.—Woman, 50 years of age, with malignant disease of thyroid, causing great dyspnœa. He was opposed to operation, but the patient insisted upon having it done. Ether was administered by the rectum. The patient died on the table before the operation was completed. Dr. Park said that this was the first case in which he had given the anæsthetics by the rectum, and it will be the last. He was almost convinced that the patient would have died of enteritis if she had survived the operation.

Case II.—Boy, 6 years of age, with acute enlargement of the thyroid gland. The swelling had compressed the trachea so that it was nearly flat, and caused great dyspnœa. During the operation, the patient became apparently moribund. By passing a tube into the trachea and artificial respiration, the patient was revived and the operation completed. The patient died of exhaustion the following day.

Dr. Bartlett suggested treating cases of goitre by leeching and venesection. He asked if the circulation of blood in the gland might not be diminished by ligating vessels, as was done in the treatment of varicose veins.

Dr. Campbell called attention to the experiments recently made by Wagner, indicating that the thyroid was an excretory gland. Wagner has operated on thirteen dogs and rabbits. When the thyroid was completely removed, the animals lived a short time in fair health. Soon they became sluggish and died in convulsions, resembling the uræmic convulsions following extirpation of the kidneys. In two cases, only half the gland was removed, and the animals continued in good health. From these experiments, Wagner concludes that the thyroid gland excretes some substance from the body, which acts as a poison to the nervous centers.

*Voluntary Communications.*—Dr. Bartlett reported a case of insomnia treated by venesection. Patient was plethoric; thirty ounces of blood removed, and a cure immediately followed.

Dr. Barnes reported a case of convulsions, with intense pain in the head, cured by drawing eight ounces of blood. Bromides and croton oil had been previously tried without beneficial effects.

*Prevailing Diseases.*—Diphtheria, sore throat, mumps and rheumatism.

